# Evaluation of the Effect of Green Tea Extract on the Prevention of Gingival Bleeding after Posterior Mandibular Teeth Extraction: A Randomized Controlled Trial

**DOI:** 10.1155/2014/857651

**Published:** 2014-06-12

**Authors:** Rasool Soltani, Abbas Haghighat, Mehrdad Fanaei, Gholamreza Asghari

**Affiliations:** ^1^Department of Clinical Pharmacy and Pharmacy Practice, School of Pharmacy and Pharmaceutical Sciences, Isfahan University of Medical Sciences, Isfahan 81746-73461, Iran; ^2^Department of Oral and Maxillofacial Surgery, School of Dentistry, Isfahan University of Medical Sciences, Isfahan, Iran; ^3^Department of Pharmacognosy, School of Pharmacy and Pharmaceutical Sciences, Isfahan University of Medical Sciences, Isfahan 81746-73461, Iran

## Abstract

*Background*. Removing tooth results in gingival bleeding. Several measures are taken to stop bleeding. In this study, the effect of green tea extract on cessation of bleeding and oozing after removing of mandibular molars was investigated. *Methods*. This was a randomized controlled clinical trial carried out on 62 patients who were referred for extraction of their mandibular molars. The volunteers were randomly and equally divided into treatment and control groups. In the first group, green tea extract-impregnated sterile gauze was used after removing the tooth while in the second group, green tea extract-free gauze was applied. Active bleeding and oozing monitoring was done every 5 minutes until cessation of bleeding and one hour after that, respectively. The results were compared using *t*-test. *Results*. The mean ± SD of bleeding duration in green tea group was significantly lower than control group (5.87 ± 1.76 versus 10.09 ± 3.61 minutes, *P* = 0.001). In addition, the number of people with oozing one hour after surgery was significantly lower in the green tea group (6 versus 29 persons, *P* = 0.001). *Conclusion*. This study showed that green tea extract contributes to significant decline in bleeding of the socket caused by tooth extraction as well as reduction of oozing.

## 1. Introduction

Gingiva is a connective and epithelial tissue that covers the jaw bone around the teeth [1]. Usually, after tooth extraction, especially molars, bleeding occurs due to creation of tooth socket. The amount of bleeding differs based on the tooth type and extent of damage to the tissue and continues in some patient for 1 to 2 days, as the consequent blood clots are unstable and removed with a partial pressure [2]. Most of the time, bleeding is controllable; however, because of bleeding complications like sluggishness, lethargy, and anemia, prevention of bleeding after tooth extraction is mandatory.

Several methods have been suggested to reduce the amount of bleeding including the use of anesthetics and materials with vasoconstrictive properties like epinephrine (1/1000), the use of chlorhexidine gel in the days before surgery to reduce the microbial load and topical inflammation [3], and the use of cellulose tampons [4] such as Surgicel tampon [5] and Gelatamp sponge [6], use of systemic preparations of tranexamic acid and vitamin K [7], and application of N-butyl-2-cyanoacrylate glue [8].

Cellulose tampons stop bleeding to an acceptable extent during 10–15 minutes, but, due to high cost, there is no public access to it [4, 5]. The so-called “Gelatamp” sponges have also shown good effect on cessation of bleeding and reduction of tooth extraction side effects [6]. However, due to having silver, it is associated with skin allergies; also, it may cause liver and respiratory complications due to silver accumulation [9]. The use of cyanoacrylate glue significantly affects the cessation of bleeding and pain after tooth extraction surgery. In addition, care should be taken for not allowing the glue to penetrate into the depth of the socket, since the body considers it as a foreign body and reacts to it resulting in delayed wound healing [8]; however, its carcinogenicity has limited its application to minor bleeding [10].

The best and most effective approach for cessation of bleeding is application of topical pressure on surgery site or consequent socket until blood clot is formed. The required time for this process is about 10–15 minutes. To prevent the blood clot from breaking, the area should not be manipulated for a certain period. The package used for applying pressure should cover whole socket area and put adequate pressure to prevent oozing [4].

In ancient days, tanniferous plants such as pomegranate, hamamelis, and tea were being used as vasoconstrictors for cessation of bleeding. The usage for menstrual and gingival bleedings is among their applications. The plant green tea (*Camellia sinensis*), which is called so due to its green color, is cultivated in most of the Asian countries with suitable climate. It contains large amounts of polyphenol compounds (with antioxidant property) and tannins [11]. The existence of tannins in this plant makes it as a potential treatment option for stopping gingival bleeding.

Given that no product is manufactured in Iran to prevent or halt gingival bleeding after surgery, this study aimed to investigate the effect of green tea extract on cessation of gingival bleeding after extraction of mandibular molars. Since this plant is largely and cost-efficiently accessible in Iran, in case of proving its effectiveness, we can recommend it as a suitable product for this purpose.

## 2. Materials and Methods

### 2.1. Preparation of Plant Extract

The green tea was extracted by percolation method, using ethanol 70%. For this, 1 kg green tea powder (Golestan, Iran) was mixed with 500 mL of alcohol 70% (Yasan, Iran). The mixture was then soaked for 24 hours for better mixing. Percolation was then performed with 5 liters of solvent. All stages of extraction process were done under sterile conditions. Using Folin-Ciocalteu method [12], the content of total phenolic compounds in the extract was determined as 18.67 mg/mL.

### 2.2. Preparation of Extract-Impregnated Sterile Gauzes

After extraction process, the sterile gauzes were soaked into the extract solution. After 1 hour, gauzes were removed from the solution by sterile forceps and pressed a little bit to remove excess extract. Then, the folded gauzes were transferred into a glass container and placed into moist heat autoclave (121°C) for 20 minutes. These medicinal gauzes were then wrapped in a sterile aluminum foil and used for clinical study. The sterilized gauzes remained moist until usage.

### 2.3. Patient Selection

Patients were selected from those who were referred for extraction of one of their mandibular molars. The inclusion criteria were (1) age ≥ 18 years, (2) no sensitivity to green tea, (3) not using complementary medical methods (herbal medicines, etc.) within the past month, (4) not having clotting disorder, (5) no acute or uncontrolled infection at the surgery site, (6) no malignancy at the surgery site, (7) no history of exposure of surgery site to radiation, (8) not using any antibiotic, corticosteroid, anticoagulant, and contraceptive drug over the past month, (9) not smoking, and (10) not having hypertension, diabetes, thyroid disorder, and blood disorder.

### 2.4. Study Design and Interventions

This was a randomized controlled clinical trial performed in Social Security Organization's dental clinic in Isfahan, Iran, in 2013. Before intervention, informed consent was obtained from all participants. The study protocol was approved by the ethical committee of Isfahan University of Medical Sciences. After inclusion of each patient into the study, his/her demographic properties (including age and gender) were recorded in the form. Patients with large periapical lesion requiring open surgery were excluded from the study. Those who met the inclusion criteria were randomly and equally assigned to treatment and control groups. Randomization was done by coin flipping. After patient selection, the desired site was anesthetized using the solution of lidocaine 2%/epinephrine 1/80000 (Darou Pakhsh, Iran) and the tooth was removed. The procedure was done as simple extraction without flap elevation, bone removal, and odontotomy. After cleansing the socket, green tea-impregnated and green tea-free sterile gauzes were applied for treatment and control groups, respectively, and the patients were asked to press the gauzes on the site. In both groups, the gauze was removed from the patient's mouth every 5 minutes, and bleeding or cessation of it was recorded. In case of bleeding, similar gauze was put on the site with pressure and the process was repeated up to one hour until bleeding stopped. The results were investigated and recorded. In addition, each patient was given a new gauze when getting discharged (after bleeding cessation) and they were asked to press it on the surgery site for one hour. After this time, by telephone contact, they were asked about the presence or absence of oozing on the site. The results were then evaluated and recorded. Finally, the mean time of bleeding cessation as well as the presence or absence of oozing was compared among the groups.

The statistical analysis was carried out by SPSS 20 using Independent-samples *t*-test.

## 3. Results

During the study, 122 patients were evaluated for inclusion into the study, among which 62 people met the desired criteria. They were then equally divided into the treatment and control groups (31 patients in each group) and were investigated. Each group was comprised of 16 males. The mean age of treatment and control groups was, in turn, 37.40 ± 11.20 and 38.51 ± 12.01 years.

The mean ± SD of bleeding time in green tea group was significantly lower than control group (5.87 ± 1.76 versus 10.09 ± 3.61 minutes, *P* = 0.001).

The number of patients who had oozing one hour after surgery was significantly lower in green tea group compared to control group (6 versus 29 persons, *P* = 0.001).

## 4. Discussion

In this study, where the effect of green tea extract on gingival bleeding after extraction of mandibular molars was investigated, most of the patients who were administered extract-free gauze (83.8%) had bleeding time of more than 5 minutes, while only 22.6% of the patients who used green tea extract-impregnated gauze experienced bleeding longer than 5 minutes. Moreover, the smaller number of patients in green tea group had postsurgery oozing; however, for better judgment about the effects of this extract on oozing, the patients are required to be monitored for longer times (e.g., 3 days). The effect of green tea on reduction of gingival bleeding and oozing, observed in this study, is probably due to its tannins which cause contraction of damaged tissue and capillaries with their astringent effect. Also, tannins have been reported to accelerate blood clotting [13]; this effect may be another possible mechanism for hemostasis. Although green tea has antioxidant and anti-inflammatory effects [14–16], the rapid decline in bleeding cannot be attributed to these effects.

To the best of our knowledge, there is no similar study about the effect of green tea on gingival bleeding. However, the effects of other plants on bleeding cessation have been investigated in a few clinical studies. In a research performed by Gupta in India, daily consumption of tablets of* Euphorbia prostrata* dry extract (containing flavonoids and tannins) by patients with grade 1 and 2 hemorrhoids significantly reduced bleeding [17]. Given the astringent effect of tannins, the use of tannic acid compounds is known among the best topical treatments for cessation of bleeding [18].

Our study shows that green tea is significantly effective for quick control of post-dental surgery bleeding. Therefore, due to easy accessibility and low price, the extract of this plant can be a good choice for stopping bleeding from the socket caused by this type of surgery. On the other hand, the effect of green tea-containing solution on decline of oral microbial load has been shown [19], which probably can help to reduce the risk of surgical site infection.

Using green tea is more cost-effective and side-effect-free compared to other topical methods such as application of laser, epinephrine, cellulose materials, and silver-containing sponge materials (Gelatamp). The laser technique is associated with a number of disadvantages like tissue damage, inflammation, and bleeding of gingival tissue [20]. The application of topical adrenaline may result in systematic disorders like cardiac complications [21]. The cellulose materials have declining impacts on collagens that exist in the tissue [22], and silver delays tissue healing and causes skin and respiratory problems due to its accumulation in the body [9].

In this study, the effect of green tea on oozing was investigated only one hour after tooth extraction, while the oozing may continue for 3 days after surgery. Therefore, performing complementary studies with different green tea products, such as mouthwash, and with longer monitoring periods is essential for evaluation of this effect.

## 5. Conclusion

The results of this study show that green tea extract is significantly effective in stopping bleeding of socket caused by tooth extraction and in reduction of consequent oozing. Therefore, by performing complementary studies, it may be found applicable in most of the clinical measures for the same purpose.
